# Ethnic and Gender Diversity in Pathology: A Dream Deferred

**DOI:** 10.7759/cureus.38528

**Published:** 2023-05-04

**Authors:** Imrana Tanvir, Amber Hassan, Shadi Alahmadi, Humaira Waseem, Javaria Anwer, Amer Shafie, Muhammad Ahmad Sheikh, Samah S Elbasateeny, Faisal Khosa

**Affiliations:** 1 Department of Pathology and Laboratory Medicine, King Abdulaziz University, Faculty of Medicine, Jeddah, SAU; 2 Translational Neuroscience Lab, CEINGE-Biotecnologie Avanzate, Naples, ITA; 3 European School of Molecular Medicine, University of Milan, Milan, ITA; 4 Department of Anatomic Pathology, King Abdulaziz University, Faculty of Medicine, Jeddah, SAU; 5 Department of Research, Fatima Jinnah Medical University, Lahore, PAK; 6 Division of Infectious Diseases, University of Louisville, Louisville, USA; 7 Department of Pathology, King Abdulaziz University, Faculty of Medicine, Rabigh, SAU; 8 Department of Financial Technology, University of Nottingham, Nottingham, GBR; 9 Department of Pathology, Zagazig University, Zagazig, EGY; 10 Department of Pathology, King Abdulaziz University, Faculty of Medicine, Jeddah, SAU; 11 Department of Radiology, Vancouver General Hospital, Vancouver, CAN

**Keywords:** gender, ethnicity, residents, diversity, pathology

## Abstract

Background

Equity, diversity, and inclusion (EDI) remain an elusive dream in the physician workforce in the United States of America (USA). Many studies have documented the tangible and intangible benefits of EDI, including the caregiver, patients, and healthcare organizations.

Objective

We aim to examine the ethnic and gender diversity trends of the active residents in pathology in United States residency programs.

Methods

A retrospective cross-sectional study was conducted on the ethnicity and gender distribution of pathology residency trainees from the academic year 2007-2018. The data was compiled from the American Association of Medical Colleges (AAMC) annual report. Data was entered and analyzed using Microsoft Excel 2013 (Microsoft Corporation, Redmond, WA, USA). Frequencies and percentages were calculated, and bar charts and pie charts were used for graphical representation.

Results

Almost 35,000 US pathology residents were enrolled according to AAMC during this particular period. The highest trend of enrolling in the field of pathology was observed in 2010 and remained the same for years. This shows that the field of pathology in the USA had some acceptance all these years. The most popular speciality in which most residents were enrolled was anatomic/clinical pathology (80%) in which females were dominant over other fields.

Conclusion

Over the years, we have failed to overcome gender and ethnicity diversity. Gender and ethnicity have a significant influence on leadership positions, academic ranks, and research productivity among pathology faculty members in the USA.

## Introduction

“Humanitarian feminine morals would help women succeed in medicine” is a well-known saying by Elizabeth Blackwell, the first medicine graduate of the United States of America (USA) who started as a medical practitioner in 1849 [1]. The USA is facing a national health crisis, as well as disparities in the quality and frequency of treatment received by racial and ethnic minorities [2]. Currently, nearly half of all medical school applicants are female, but only 39% of women have full-time academic faculty positions. There is still a significant global gender disparity in the scientific community, and females are regarded as less capable leaders than males in academic medicine. Women are underrepresented in several leadership positions, despite having the same prevalence as male faculty [3]. The population trend of non-White (minority) people in the USA was 39.6% in 2010, and by 2020, it raised to 50.2% [4]. Although Black, Hispanic, and Native Americans constitute one-third of the US population, they account for only 9% of practicing physicians in the country, which has not changed significantly over the last 30 years [5]. In this population, which is becoming more diverse than any other part of the world, racially diverse physicians can help us cope and provide greater access to healthcare [6]. A 2016 survey reported that pathology has the third greatest proportion of women to men of any medical speciality (42%) [7]. Lett et al. [8] found statistically significant trends of increasing underrepresentation of Blacks and Hispanics (both genders) in nearly all specialities (including pathology).

Through an interactive process, physician gender influences the patient-physician relationship [9]. Although there is no significant interaction between pathologists and patients, Pathology Explanation Clinics promise to provide patients with better-explained and understanding problems and solutions to facilitate decision-making [10]. Female physicians had a higher rate of patients of breast and cervical cancer screening because of differences in beliefs and patient preference for a female provider [11]. Significant studies demonstrated the importance of diversity in the medical field, and there were studies demonstrating the underrepresentation of certain ethnic groups or genders. Llorens et al. [12] concluded that women are underrepresented in senior academic ranks and leadership in psychiatry, despite accounting for 42% of faculty members.

Pathology has the third highest proportion of women to men among all medical specialities (42%), according to recent data for 2021. The gender disparity studies focused on the departmental need to diversify the criteria for recruitment to select high-potential women for future leaders and cultivate the benefits of diversity [12].

The purpose of this study was to assess gender disparities in academic pathology in the USA at various levels of leadership, academic rank, and publication output, which are common academic criteria that can influence leadership selection. Furthermore, the domain of pathology lacks a comprehensive study examining gender differences, particularly in academic achievements. It is essential to comprehend that diversity entails the inclusion of people from all walks of life, including backgrounds, races, religions, genders, socioeconomic statuses, and abilities. Diversity is not just different people; it is also different perspectives, approaches, and worlds in one room.

## Materials and methods

A retrospective cross-sectional study of academic pathology faculty members was conducted. Our method has been validated in several recent publications that used the same definitions and data collection process [13]. Because the data were all available in the public domain and there were no patient confidentiality concerns, no institutional review board approval was required.

Study population

The data summarizes the characteristics, using gender and ethnicity as variables to quantify diversity among the active residents in pathology and its subspecialties in US residency programs. The data was extracted from the Accreditation Data System (ADS) via the Accreditation Council for Graduate Medical Education (ACGME) data resource books from the 2007-2018 academic year. At the start of each academic year, programs update the system with information on new incoming residents. ADS is currently the most comprehensive database of ACGME Sponsoring Institutions, programs, and physicians-in-training in the United States. Trainees were categorized into the following ethnic groups: White (non-Hispanic), Asian or Pacific Islander, Hispanic, Black (non-Hispanic), and others not included in previously mentioned ethnic groups such as Native Americans/Alaskans and unknown (unreported). Gender was characterized as male and female. First, we assessed the trends in ethnic diversity in pathology residents between the academic years 2007 and 2018 by examining the changes in the proportions of White, Asian, Hispanic, and Black residents as well as gender (male versus female). We also compared the racial and gender distribution in pathology by graphical representation according to the tenure track system, academic ranks, and degree status from 2007 to 2018. There was no data on ethnic diversity before 2007 to be assessed.

Similarly, we examined the trends in gender diversity from 2007 to 2018 by examining the changes in the proportions of female trainees. We then compared the proportions of female trainees in pathology from 2007 to 2018. We included all pathology faculty who held a university academic rank and had the equivalent degree of Doctor of Medicine (MD) (i.e., Doctorate of Medicine and of Philosophy (MD-PhD) or Doctors of Osteopathic Medicine (DO)). We divided academic rank into four categories based on published studies and hierarchy: professor, associate professor, assistant professor, and instructor/lecturer. We separated the data for leadership rank into the highest level: any of chairpersons, heads, deans, program directors, or residency program directors; second highest level: any vice president, deputy director, or vice dean; and no leadership role. Faculty without an MD equivalent degree, emeritus professors, and administrative staff members were excluded.

Statistical analysis

Data was entered and analyzed using Microsoft Excel 2013 (Microsoft Corporation, Redmond, WA, USA). Frequencies and percentages were calculated, and bar charts and pie charts were used for graphical representation.

## Results

**Figure 1:**
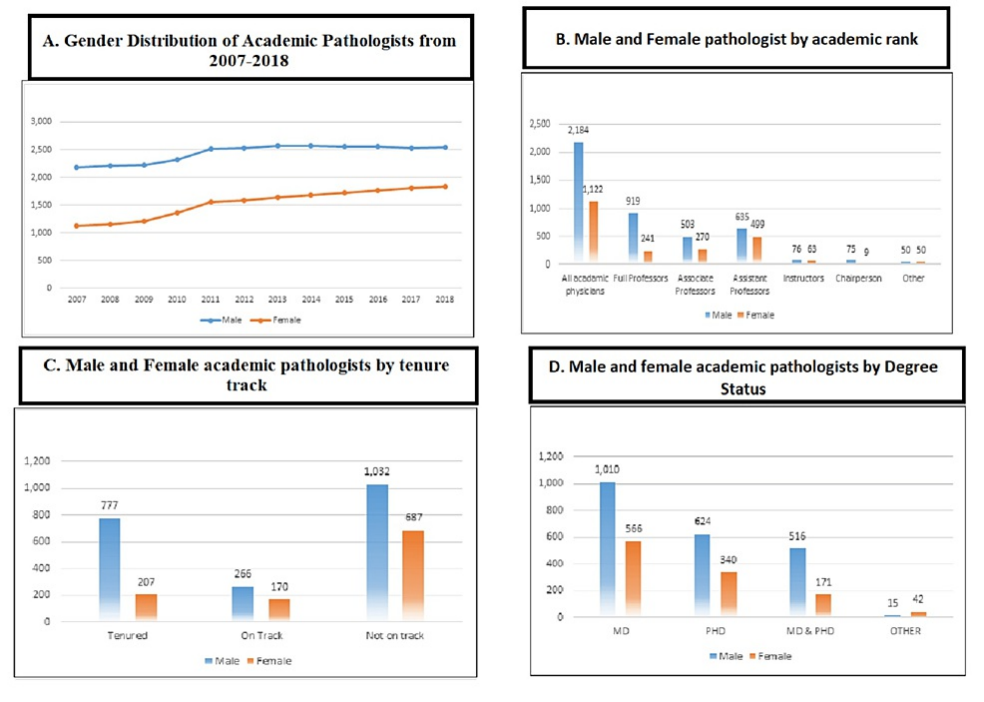
Gender distribution of academic pathologist positions from 2007 to 2018 (A) with respect to academic rank (B), tenure track (C), and degree status (D).

Our study includes a total of 47,736 pathologists and pathology residents enrolled from 2007 to 2018. Among them, 41.99% were male and 59.23% were female. When the absolute change was observed, it was determined that from 2007 to 2018, the female ratio was changed by 5.13% versus male by 2.82%, which shows a significant increase in females. The gender distribution of academic pathologists showed that the majority of pathologists did not have a tenure track, of which 1,032 were male and 687 were female. A comparison of 2007-2018 tenure track data showed that the ratio of males was higher than females for tenured (777 versus 207), on track (266 versus 170), and not on tenure (49 versus 28). Our data represented the high frequency of male gender compared to female according to the higher degree status such as in MD degree (1,010 versus 566), PhD (624 versus 340), MD-PhD (516 versus 171), and others (15 versus 42). We also compared our data for academic ranks in terms of gender: male versus female for all academics (2,184 versus 1,122), full professors (919 versus 241), associate professors (503 versus 270), assistant professors (635 versus 499), lecturers (76 versus 63), and department chairs (75 versus 9). Our data indicated that the predominance of the male gender in the field of pathology was reported as 41.99%, and 1.62% had a tenured position. Among them, 0.211% had a graduate degree (MD) and were academic physicians (4.57%) (Figure 1).

**Figure 2:**
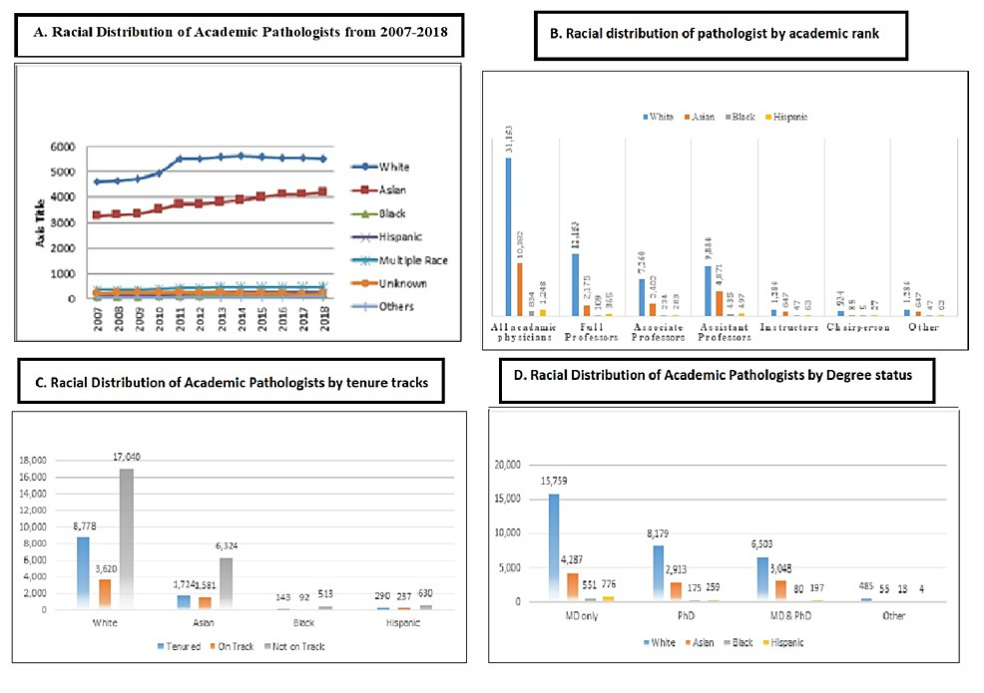
Racial distribution of academic pathologist positions from 2007 to 2018 (A) with respect to academic rank (B), tenure track (C), and degree status (D).

When data were compared for racial distribution, White was in the majority (65.2%), followed by Asian (21.7%), multiple races (5.31%), unknown (2.63%), Hispanic (2.61%), and others (0.67%). The absolute change from 2007 to 2018 was highest in White (65%), followed by Asian (3.2%), multiple races (0.54%), unknown (0.19%), Hispanic (0.27%), and others (0.36%). The highest trend was observed in 2010 and remained the same for years. This shows that the field of pathology in the USA has been accepted all these years. When data were compared for racial distribution from 2007 to 2018 according to tenure track status, it was observed that a maximum number of pathologists were White, followed by Asian, Black, and Hispanic. Regarding complete tenure versus on track, the majority was White (8,778 versus 3,620), followed by Asian (1,724 versus 1,582), Hispanic (290 versus 237), and Black (143 versus 92). The data were also compared according to degree status among racial disparities, which show that White has the highest degree in MD (15,759), followed by Asian (4,287), Hispanic (776), and Black (551). Regarding academic rank, the same pattern was seen. White (31,153) was in the first position in all academic ranks, followed by Asian (10,382), Hispanic (1,248), and Black (834). Maximum seats of chairpersons were held by White. It was observed that Black was lesser in number concerning diversity. White shows dominance in all categories of diversity in pathology (Figure 2).

## Discussion

We have seen that over time, the percentage of female residents has increased. Our data give us an idea of the pathology workforce working in the hospitals now. In the current study, the most popular trending field of pathologists and its difference concerning gender during 2007-2018 were determined. Almost 35,000 US pathologist residents were enrolled according to AAMC during this particular period. The highest trend was observed in 2010 and remained the same for years. This shows that the field of pathology in the USA has been accepted all these years. Males exceeded females in higher-ranking jobs such as chairperson, full professor, and associate professor, but females outnumbered males in lower-ranking positions such as assistant professor and instructor (Figure 1). Women continue to face barriers to academic advancement in medical positions; promoting and supporting women in senior roles, increasing mentorship opportunities from women in leadership positions, and developing more family-friendly career paths with flexible or part-time commitments are needed.

As the ranks decreased in the hierarchy, other races increased in representation. Blacks and Hispanics comprised only 2.7% of academic pathologists with negligible increases in representation over the past 12 years (Figure 2). Since 2007, only 5,728 (16.25%) residents were enrolled, which increased to 5,976 (16.95%) in 2010, followed by the same trend from 2012 to 2018. A study conducted in France on gender-based diversity shows that between 1989 and 2015, 3,950 professors were appointed, of whom one in five were women. Female professors consistently represented a minority in all French region’s specialities over the study period. Although a small increase was observed over the years, women never represented more than 29% of newly appointed professors [13]. The finding of this study contradicts the current study as females in the field of pathology were more dominant. It was also observed that in a medical setting, women have been shown to spend more time on patient care and teaching than men.

Another study conducted on sex differences in workplace satisfaction and engagement of academic pathologists in the USA showed that women report more time in patient care and less time in research. Women consider formal mentorship, feedback, and career advancement more important than men do and are less satisfied with communication and governance. The Association of Pathology Chairs (APC) survey showed that 20%-40% of non-chairperson department leaders were women. More than half of the departmental positions report satisfaction with the gender diversity of their departmental leaders [14,15]. Of academic pathology departments, 15.4% are chaired by the women’s physician workforce. The data available across the specialities reports a 7.2% decrease in active physicians practicing anatomic and clinical pathology in the 2012 AAMC Specialty Data Book [16]. In the study, it was reported that in 2012, there were 16,835 medical school graduates, among them 48.3% were female, and 15.3% were of underrepresented minorities (URMs) (Blacks, Hispanics, and American Indians (AIs), Alaska Natives (ANs), Native Hawaiians (NHs), and Pacific Islanders (PIs)) [17].

Numerous studies showed that, as organizational leaders, women are not only as competent as males but also are more capable than many males in generating work-life balance and implementing change and quality improvements in any system. Women doctors communicate better with patients and colleagues and take less risk than their male colleagues. Female physicians spend more time with their patients, are more likely to engage their patients in discussions of their social and psychological context, and deal more often with feelings and emotions [18]. Female physicians facilitate partnership and patient participation in the medical exchange more effectively than male physicians. The quality of the interactive process is critical to the establishment of a therapeutic relationship, and this process is related to the physician’s gender [19]. However, McKinstry disagreed: “Empathy and communication skills are important, but so are efficiency and the ability to live with risk” [20,21].

Based on that, academic pathologists can make strategic plans to develop a culture of flexibility in the field. As a result, they can draw medical students to the discipline while also mentoring and inspiring female academics to pursue top academic and leadership positions.

## Conclusions

Gender and ethnicity have a significant influence on leadership positions, academic ranks, and research productivity among pathology faculty members in the USA. In this study, we examined the influences on gender disparity among US academic pathologists using commonly cited measurable factors. It would be beneficial to conduct detailed studies on variables such as marital status, number of children, age of faculty members at various stages of academic rank or position, and impact on mentees. Women pathologists were well represented at the assistant professor/instructor academic rank but underrepresented at the professorial rank, with the differences being more pronounced. A similar disparity was observed in positions of leadership.
